# Editorial: Pediatric Inflammatory Bowel Diseases: Looking to the Future

**DOI:** 10.3389/fped.2020.00056

**Published:** 2020-02-26

**Authors:** Eytan Wine, Wael El-Matary, Jeff Critch, Víctor Manuel Navas-López, Seamus Hussey

**Affiliations:** ^1^Division of Pediatric Gastroenterology, Departments of Pediatrics and Physiology, University of Alberta, Edmonton, AB, Canada; ^2^Section of Pediatric Gastroenterology, Department of Pediatrics, Max Rady College of Medicine, University of Manitoba, Winnipeg, MB, Canada; ^3^Department of Pediatrics, Memorial University, St. John's, NL, Canada; ^4^Pediatric Gastroenterology and Nutrition Unit, Hospital Regional Universitario de Málaga, Málaga, Spain; ^5^National Children's Research Centre, University College Dublin (UCD), Dublin, Ireland; ^6^Royal College of Surgeons in Ireland (RCSI), Dublin, Ireland

**Keywords:** inflammatory bowel disease, Crohn disease, ulcerative colitis, pediatrics, editorial, Research Topic

The incidence of inflammatory bowel diseases (IBD) is increasing across the globe (1), especially in children (2). This highlights not only the clinical importance of characterizing pediatric IBD (P-IBD) but also provides an opportunity to improve understanding of pathogenesis and patient outcomes. In this editorial, 12 manuscripts are reviewed, all published in the *Research Topic: Pediatric Inflammatory Bowel Diseases: Looking to the Future*. These papers span a diverse array of topics related to P-IBD, from rare complications, through evolving assessment tools and clinical features, to novel pathways, possibly leading to future treatments. These publications focus on features of IBD unique to children, and will serve as a resource for anyone interested in P-IBD.

Clinical expertise incorporates vigilance for recognizing unusual clinical patterns and pursuing appropriate differential diagnoses in such circumstances. Lawrence et al. reported on a 12-year-old girl with Crohn disease who presented with a short history of progressive respiratory distress and hypoxemia, requiring intensive respiratory support. Cardinal factors in this patient's case included profound lymphocytopenia, thiopurine immunosuppression, recent corticosteroid exposure, chest radiographic findings, and a sputum sample for direct microbial testing. Following a diagnosis of *Pneumocystis jirovecii* pneumonia (PJP), targeted therapy was successful. Current pediatric guidelines recommend considering PJP prophylaxis for patients on triple immunosuppression that includes corticosteroid therapy (3).

Collins et al. described a teenager with relapsing Crohn disease whose weight had fallen from the 25th to the 3rd percentile. Weight loss continued in hospital while on full exclusive enteral nutrition (EEN) of 2,400 kcal/day. Weight gain followed a period of 24-h nursing observation. Psychiatric evaluation led to a diagnosis of anorexia nervosa. The sentinel clinical feature was weight loss despite apparently high calorie intake. Anorexia nervosa, rather than Crohn disease, is more often considered as the potential cause of weight loss in young adults. The coexistence of both diagnoses may be associated with poor outcomes (4).

Acute neurological complications in children with IBD are rare, but should prompt consideration of intracranial vascular thrombosis, especially in the setting of active disease and thrombosis risk factors. Martín-Masot et al. describe a 5-year-old patient with refractory ulcerative colitis (UC), who developed a focal headache, upper limb paresis, and seizures within 4 days of colectomy and J-pouch creation. Potential risk factors in this case included active preoperative disease resulting in reduced mobility, recent major abdomino-pelvic surgery, and central venous catheter placement. The patient had not been on thromboprophylaxis therapy during the perioperative period. A recent Pediatric clinical guideline recommends thromboprophylaxis in high risk patients, following individual risk assessment (3).

Disease aspects unique to P-IBD are discussed in three additional papers included in this *Research Topic*. Acute severe UC (ASUC) is a medical emergency. Pediatric-onset UC is usually more extensive and follows a more progressive course than in adults (5). Since disease severity has consistently been associated with disease extent, children are especially susceptible to severe refractory flares. With few exceptions, children with ASUC should be admitted to the hospital for intensive medical treatment. Akintimehin et al. published the results of the first population-based study of ASUC in Irish children. In this retrospective study of 55 children >41% required colectomy. Colectomy rates were higher in the pre-infliximab period (88 vs. 36%). The authors did not identify predictive factors of response to infliximab.

Endoscopy is the cornerstone in assessing mucosal healing in IBD. However, this is a relatively invasive and costly procedure. This has led to the search for non-invasive and less expensive markers of intestinal inflammation. In a single center, retrospective cohort study, El-Matary et al. examined the impact of measuring fecal calprotectin (FCAL), a stool marker for intestinal inflammation, on decision-making and clinical care of children with IBD. The majority (86%) of positive FCAL measurements (≥250 μg/g of stools) resulted in treatment adjustments with subsequent clinical improvement. Conversely, 83% of those with normal FCAL remained in clinical remission over the following months without any changes in medical management. This study was limited by its retrospective design, small sample size, and limited data correlating mucosal healing with FCAL. In their commentary article, Day and Adamji highlighted the benefit of non-invasive stool markers in monitoring IBD. They proposed that serial FCAL measurements may be a more effective strategy to inform individual patient care than a one-off level.

Extraintestinal manifestations (EIM) occur in ~1/3 of P-IBD cases. Pancreatic disease is a less frequently observed EIM, potentially manifesting in multiple ways, as highlighted by Martin-de-Carpi et al. Pancreatic involvement can present as acute pancreatitis, chronic pancreatitis, autoimmune pancreatitis, asymptomatic exocrine pancreatic insufficiency, increased pancreatic enzyme levels, structural abnormalities, and granulomatous inflammation, as well as therapy-associated pancreatitis. Clinicians caring for children with IBD should be familiar with these conditions, diagnostic considerations, and treatment options.

In a separate article, Day and Brown present their experience with using EEN, as an adjunctive therapy, in two cases with Crohn disease complicated by phlegmon to avoid resection, at least in the short term. EEN is recognized as an effective induction agent for active luminal inflammatory disease (6), but in this situation its use in complicated Crohn disease may have prevented the need for bowel resection.

This last section focuses on emerging concepts and potential pathways for future therapies in P-IBD. Multiple factors have been related to the pathogenesis of IBD, including genetic mutations, intestinal immune dysfunction, impaired intestinal microbiota, dietary factors, antibiotics, infections, type of delivery, appendectomy, urban environment, smoking, sleep disorders, and stress. Sun et al. reviewed the role of stress in IBD, explaining in detail the mechanisms by which stress affects IBD in relation to homeostasis and gut motility, intestinal microbiota, and immune and neuroendocrine dysfunction. Stress exerts a deleterious effect at different stages of IBD (onset, disease course, and prognosis). This review highlights that mechanisms related to stress should be considered as a strategy both in prevention and in the treatment of IBD.

Surgical resection is often indicated for complicated fibrostenotic and/or internally penetrating Crohn disease. Recent efforts have focused on more completely controlling inflammation to prevent disease progression and hopefully decrease the need for surgery. As Stenke et al. discuss, while no antifibrotic therapies are currently available for Crohn disease, recent research opens the possibility that targeting the profibrotic transforming growth factor-B (TGF-B) may lead to effective antifibrotic therapies in the future. This may lead to potential therapeutic options to mitigate the effects of fibrosis and reduce the need for surgical intervention.

Another key pathway, well-recognized in the pathogenesis of IBD, involves nuclear factor kappa-light-chain-enhancer of activated B cells (NF-κB), a nuclear transcription factor known to activate pro-inflammatory pathways. Novel approaches to regulate NF-κB and its downstream consequences are reviewed by Zaidi and Wine; these relate to recent discovery of pediatric-specific changes in A20, which is a key regulator of NF-κB, as shown in samples from children with IBD (7). The authors propose that instead of suppressing inflammation, efforts should be made to regulate key pro-inflammatory pathways, thus preventing uncontrolled inflammation to begin with.

Finally, given the recent attention to food and food additives in the pathogenesis of IBD (8), the unique role of carrageenan and carboxymethylcellulose is reviewed by Martino et al. Both chemicals are commonly-used emulsifiers and food stabilizers. Much of the evidence on the effects of these additives on gut health, and how this might relate to IBD, is based on animal studies, but the relevance of these mechanisms to human IBD is reflected in the utility of new nutritional approaches, such as the Crohn-disease exclusion diet (CDED) (9).

Together, this collection of reports presents recent advances in our understanding of P-IBD and opportunities for improving care through further research and innovation.

## Author Contributions

All authors listed have made a substantial, direct and intellectual contribution to the work, and approved it for publication.

### Conflict of Interest

The authors declare that the research was conducted in the absence of any commercial or financial relationships that could be construed as a potential conflict of interest.
